# Optimal Timing for Intraocular Pressure Measurement Following Phacoemulsification Cataract Surgery: A Systematic Review and a Meta-Analysis

**DOI:** 10.3390/vision8040065

**Published:** 2024-11-08

**Authors:** William J. Herspiegel, Brian E. Yu, Hamzah S. Algodi, Monali S. Malvankar-Mehta, Cindy M. L. Hutnik

**Affiliations:** 1 Schulich School of Medicine & Dentistry, Western University, London, ON N6A 3K7, Canada; byu2026@meds.uwo.ca (B.E.Y.); halgodi@uwo.ca (H.S.A.); monali.malvankar@schulich.uwo.ca (M.S.M.-M.); cindy.hutnik@sjhc.london.on.ca (C.M.L.H.); 2 Ivey Eye Institute, St. Joseph’s Health Care, London, ON N6A 4V2, Canada

**Keywords:** cataract, intraocular pressure, phacoemulsification, cataract surgery, cataract extraction, post-operation, timing

## Abstract

Post-operative increases in intraocular pressure (IOP) are a frequent complication following phacoemulsification cataract surgery. Assessment of IOP is an essential element in post-operative checks. Despite this, guidance regarding the optimal timing remains vague. The purpose of this meta-analysis was to determine the current status of evidence that may help guide best practice regarding the optimal time following phacoemulsification cataract surgery to measure IOP. A comprehensive literature search was performed on MEDLINE and EMBASE. In two stages, independent reviewers screened articles that reported IOP measurements following uncomplicated cataract surgery. Risk of Bias Assessment was conducted following data extraction. The meta-analysis incorporated 57 randomized clinical studies involving a total of 6318 participants and 7089 eyes. Post-operative hour one had a significant decrease in IOP from baseline, while hour two had a non-significant increase. Post-operative hours four, six, and eight were the only timepoints to have a significant increase in IOP. Finally, post-operative day one had no significant change in IOP, while day two had a non-significant decrease. These results suggest that the optimal time to measure IOP is within the first 4–8 h following phacoemulsification cataract extraction. Taking measurements too soon or too late could result in missed IOP spikes.

## 1. Introduction

Cataractogenesis is a normal physiological change associated with aging and is the leading cause of reversible visual impairment worldwide, affecting 95 million people globally and 3.5 million Canadians [1,2]. This incidence is expected to increase as both North America’s and the world’s population continues to age [3,4]. Through surgical intervention, cataracts can be treated with minimal complications [1]. Consequently, cataract extraction surgery has become a prevalent procedure and is projected to experience significant growth, both in North America and worldwide [3,5].

Phacoemulsification is the primary method for cataract removal in developed countries. It offers several advantages, including smaller incisions, less tissue damage, and a lower risk of complications after surgery [6,7].

A common complication of cataract surgery is elevation of intraocular pressure (IOP). This can occur in both expert and novice hands, largely due to the incomplete aspiration of viscoelastic materials used to facilitate the extraction [8]. These IOP spikes can result in increased morbidity in the acute period immediately following, and up to one month after, cataract surgery [9,10]. This includes pain, nausea, vomiting, corneal edema, and blurred vision, which can all result in the patient contacting the operating surgeon or being brought to the emergency room [9,10,11,12]. Additionally, in eyes with pre-existing optic nerve damage, such as those with glaucoma or atherosclerosis-related ischemia, IOP spikes can cause worsening of these sight-threatening diseases [12].

The American Academy of Ophthalmology (AAO) provided guidelines regarding the optimal timing of IOP measurements post-surgery in the “2021 Cataract in the Adult Eye Preferred Practice Patterns” [6]. The AAO recommended that IOP should be measured within the first 48 h after surgery in patients with low-risk surgeries and with no signs or symptoms of possible complications following cataract surgery. Additionally, these preferred practice patterns recommend that functionally monocular patients and those at high risk of early postoperative complications should have their IOP measured within the first 24 h after surgery. More specific recommendations regarding the exact timing are not indicated. The timing has thus become more a matter of convenience for the surgeon rather than when the undesired outcome is most likely to be detected. The lack of more specific guidance likely accounts for the variability of IOP measurements noted within the first 48 h post-surgery.

The purpose of this meta-analysis was to review and synthesize the available evidence to determine if there is an optimal period following phacoemulsification cataract surgery to measure IOP. The goal was to help guide surgeons and healthcare systems regarding best practice.

## 2. Materials and Methods

### 2.1. Search Strategy

A literature search was conducted in the following two databases: MEDLINE and EMBASE. The search had no limitations placed on publication date and, therefore, included all studies published before 7 February 2023. Sets of keywords relating to Intraocular Pressure, after (i.e., post, following), and Cataract Surgery (i.e., cataract surgery, cataract surgeries) were used with restrictions placed on adult human subjects, English-published literature, and randomized clinical trials. All articles were then imported into Covidence (Veritas Health Innovation, n.d.), which is a web-based systematic review screening tool that was used to remove duplicates and create two levels of screening: title and abstract screening and full-text screening.

After all articles had been imported into Covidence, two reviewers independently screened titles and abstracts for articles that measured IOP following uncomplicated cataract surgery. Articles that were accepted past the first level of screening then proceeded through a second level of screening where three reviewers independently screened full texts for publications that accurately measured IOP following uncomplicated phacoemulsification cataract surgery. Conflicts at both levels of screening were resolved through discussion to find a consensus between the reviewers (WJH, BEY, and HSA). In cases where consensus was not achieved, the third reviewer was used to provide a decision. After each screening level, chance-corrected kappa statistic was used to assess interobserver agreement for the inclusion of studies. This study’s detailed protocol was registered through Inplasy (Registration number: INPLASY202490004).

### 2.2. Inclusion and Exclusion Criteria

The population of interest consisted of human adults who, following routine cataract surgery, had their intraocular pressure measured. First, included publications were required to report a measurement of IOP through tonometry and after completion of cataract surgery. Second, at least one IOP measurement had to have taken place at three different timepoints: at baseline before cataract surgery, within the first 48 h following cataract surgery, and after these first 48 h. The timeline criteria are in line with 2021 Cataract in the Adult Eye Preferred Practice Patterns [6]. Third, the IOP measurement outside the first 48 h was used to ensure IOP did decrease relative to baseline after this acute rise. Studies were included if the cataract extraction method of choice was phacoemulsification. Fourth, studies that examined participants who, in addition to cataracts, also had glaucoma and pseudoexfoliation syndrome (PXF) were included. Lastly, only randomized clinical trials published in English were considered for this review.

Studies were excluded if the cataract surgery was combined with another ophthalmologic surgery. Publications that focused on participants younger than 18 or had an ophthalmologic condition other than cataract or glaucoma were excluded. Exclusion criteria were nonhuman and non-English studies. Studies that reported IOP data in figure form that could not be accurately determined were also excluded. No limits were placed on study location, publication date, or sex.

### 2.3. Risk of Bias Assessment

The quality of each study was assessed using the CLARITY risk of bias instrument for randomized controlled trials [13]. This assessment tool measures the risk of bias based on six factors: (1) adequacy of allocation sequence generation, (2) adequacy of allocation concealment, (3) study blinding, which is further subdivided into 3a. patient blinding, 3b. healthcare provider blinding, 3c. data collectors blinding, 3d. outcome assessors blinding, 3e. data analysts blinding, (4) frequency of missing outcome data lost during follow-up, (5) degree of selective outcome reporting, and (6) Other potential problems that could put the study at risk of bias. For missing data, various pieces of available information (such as the range, *p*-value, and confidence interval) were utilized and converted to the common effect measure.

### 2.4. Data Extraction

For each included study, quantitative and qualitative information related to participants’ IOP was collected independently. The following data were extracted: study information (i.e., author and year), study characteristics (i.e., number of participants, number of eyes examined, mean age of participants, sex of participants, location of study, disease population of interest, study blinding, and corporate funding), study surgical parameters (i.e., Ophthalmic Viscosurgical Device (OVD) used, method of phacoemulsification, and pre-operative, intra-operative, and postoperative medications used), and studies IOP specific characteristics (i.e., time of IOP measurement, pre-operative baseline IOP measurement, all IOP measurements within the first 48 h post-cataract surgery, one IOP measurement outside these first 48 h, method of IOP measurement, patient’s position during IOP measurement, and any IOP measurement corrections related to clear corneal thickness measurements). Data entry was extracted manually from studies into an Excel sheet. Further, corresponding authors were also contacted for additional information.

### 2.5. Statistical Analysis

Meta-analysis was completed using STATA v. 18.0 (STATA Corporation, 2023, College Station, TX, USA). The main outcomes of interest were the mean and standard deviation (SD) of pre- and post-operative IOP. For change in IOP, the standardized mean difference (SMD) was calculated as the mean difference in IOP from baseline. To test heterogeneity, statistics, Z-value, and χ^2^ statistics were computed. A value of less than 50% implied low heterogeneity, and, in these cases, a fixed-effect model was computed. Statistics of 50% or more represented high heterogeneity, and, in these cases, a random-effect model was calculated. Additionally, a high Z-value, a low *p*-value (<0.01), and a large value implied significant heterogeneity, and, therefore, a random-effect model using DerSimonian and Laird methods was computed. This study adopted a significance level of 0.05. Forest plots were also generated for each case. Funnel plots were generated to check publication bias. If funnel plots were found to be asymmetrical, standard Egger’s regression test for small-study effects and the Trim and Fill method were performed to further check for publication bias. Causes of heterogeneity were also explored.

## 3. Results

### 3.1. Search Results

**Figure 1:**
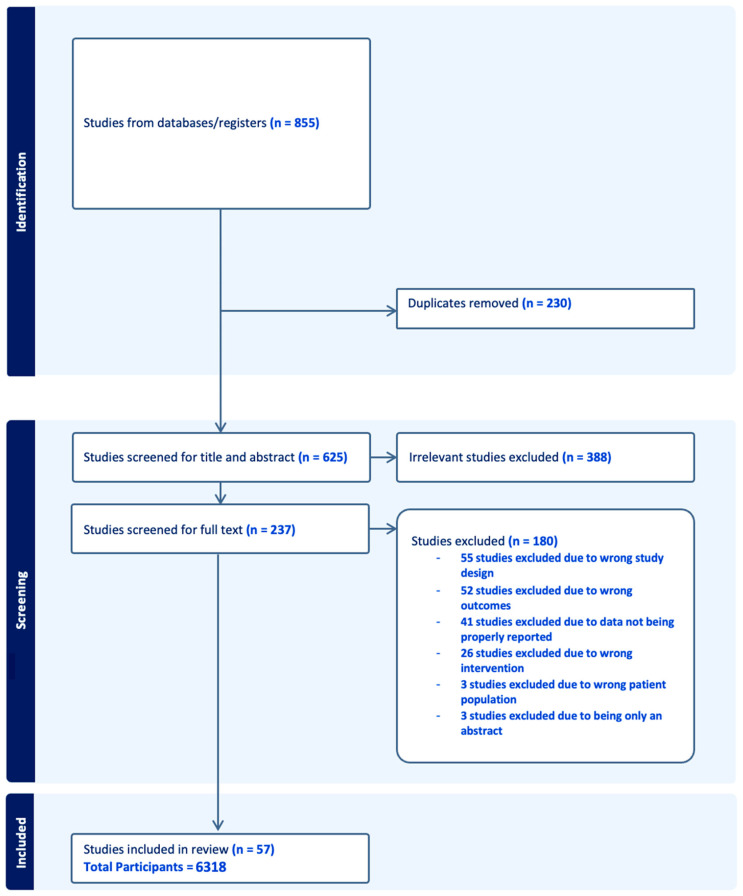
PRISMA flow diagram for optimal timing for intraocular pressure measurement following phacoemulsification cataract surgery.

Database searches resulted in 855 published literature records. The 855 records were then imported into the Covidence systematic review software, version 2.0, with 230 duplicates being removed. At the end of the title and abstract screening, 237 articles remained that moved on to the full-text screening. After the full-text screening, 57 randomized clinical trial studies were included. The Kappa statistic for the first and second levels of screening came to 0.439 and 0.710, respectively. The meta-analysis study retrieval process is detailed in a Preferred Reporting Items for Systematic Reviews and Meta-Analysis (PRISMA) flow diagram (Figure 1) [14].

### 3.2. Study Characteristics

This meta-analysis included 57 randomized clinical trial studies with a total of 6318 participants and 7089 eyes (Appendix A Table A1) [15,16,17,18,19,20,21,22,23,24,25,26,27,28,29,30,31,32,33,34,35,36,37,38,39,40,41,42,43,44,45,46,47,48,49,50,51,52,53,54,55,56,57,58,59,60,61,62,63,64,65,66,67,68,69,70,71]. A total of 43.7% of the included participants were male, and the mean age of included participants was 68.4 years old. Four studies did not report the number of male participants. Two studies did not report the mean age of participants. Out of all the studies, 12 were performed in Austria, nine in Turkey, five in Germany, four in the United States, two in Brazil, two in Canada, two in China, two in India, two in Italy, two in Japan, two in Netherlands, one in Czech Republic, one in Finland, one in France, one in Greece, one in Iran, one in Israel, one in Korea, one in Pakistan, one in Poland, one in Spain, one in Sweden, and one in Thailand. In addition, there was one multicentre study in a combination of European countries and one multicentre study across Canada, the United States, Germany, Italy, Mexico, the Philippines, Poland, Spain, and the United Kingdom. Studies’ publication dates ranged from 1992 to 2023, with 65.5% of studies taking place in the last 20 years. A total of 52 studies included patients with only cataracts, two studies looked at cataract patients with glaucoma [29,70], one study looked at cataract patients with pseudoexfoliation syndrome (PEX) [15], one study looked at both patients with only cataract and cataract with PEX [31], and one study looked at patients with only cataract, cataract with glaucoma, and cataract with PEX [40].

### 3.3. Risk of Bias Assessment Results

Out of the 57 included studies, 10 were considered at a high risk of bias [23,26,27,30,34,35,36,47], 21 were considered to have some concerns of bias, and 26 were considered at low risk of bias (Appendix A Table A2). The concerns stemmed from blinding, as 7 studies were open-label, and 3 studies were single-blinded, while 15 studies had unclear information surrounding blinding. Another cause for concern was unclear information about allocation sequence generation and concealment in 31 studies.

### 3.4. Publication Bias

**Figure 2:**
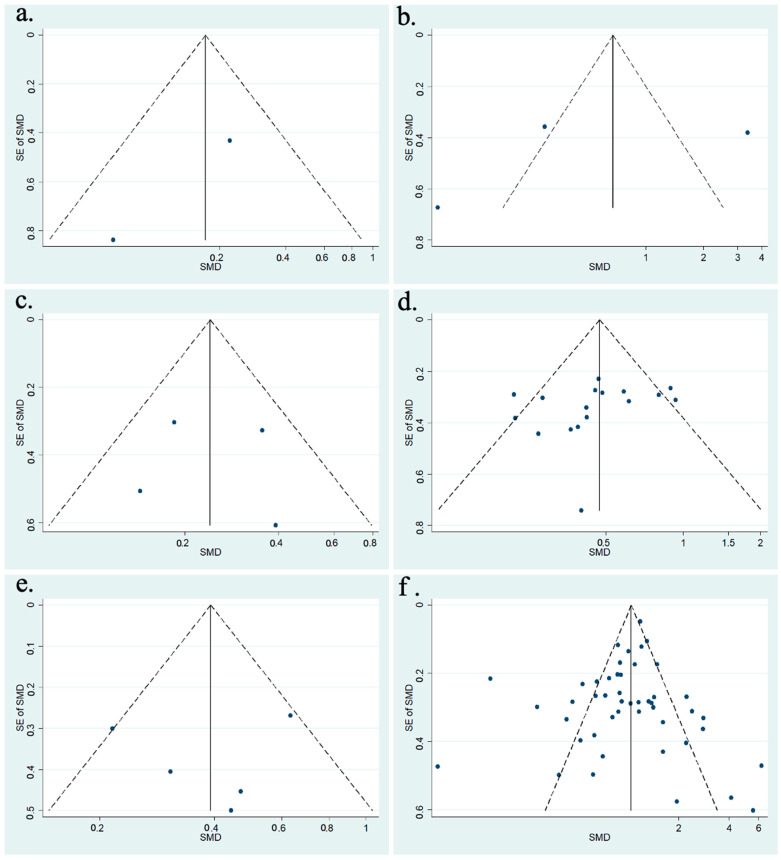
(**a**) Funnel plot for included meta-analysis studies that provided data on the change in IOP from baseline at 1 h following phacoemulsification; (**b**) funnel plot for included meta-analysis studies that provided data on the change in IOP from baseline at 2 h following phacoemulsification; (**c**) funnel plot for included meta-analysis studies that provided data on the change in IOP from baseline at 4 h following phacoemulsification; (**d**) funnel plot for included meta-analysis studies that provided data on the change in IOP from baseline at 6 h following phacoemulsification; (**e**) funnel plot for included meta-analysis studies that provided data on the change in IOP from baseline at 8 h following phacoemulsification; (**f**) funnel plot for included meta-analysis studies that provided data on the change in IOP from baseline at 1-day following phacoemulsification.

To assess the risk of publication bias, a funnel plot was generated for every hour IOP was measured following cataract surgery. Figure 2 shows the funnel plot for studies that provided data on the change in IOP for hours 1, 2, 4, 6, and 8, as well as day 1 following phacoemulsification. Included studies for post-operative hours 1, 2, 4, and 8 are scattered symmetrically on either side of the funnel plot; however, there were only a few data points. The funnel plot was symmetrical, but no conclusions could be made due to a smaller sample of studies that took IOP measurements at these timepoints. The funnel plot for post-operative hour 6 had many studies scattered symmetrically along the top of the plot, with fewer along the middle and bottom portions, indicating there were fewer small-scale studies included. The included studies for post-operative day 1 were scattered symmetrically on either side of the plot, although some fell outside the 95% confidence interval. Although the funnel plot at this timepoint appeared symmetrical, it is important to note that this was only one indicator of publication bias.

**Figure 3:**
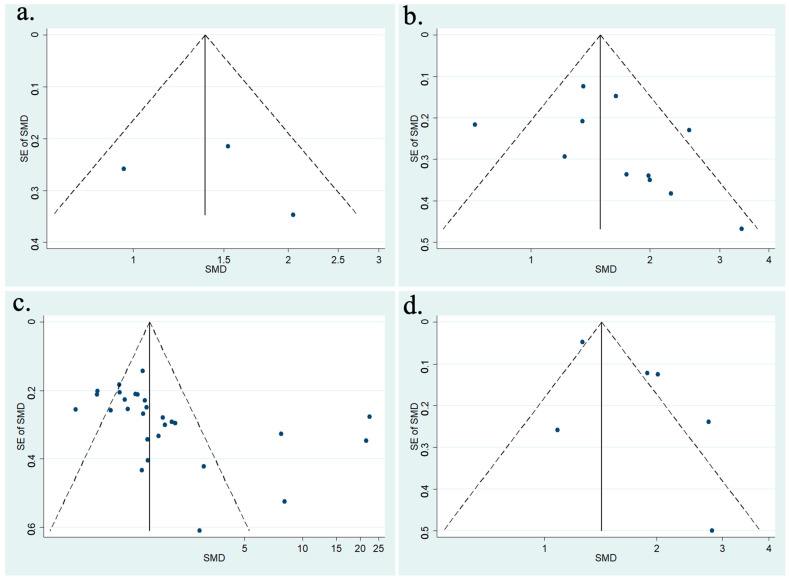
(**a**) Funnel plot for included meta-analysis studies that provided data on the change in IOP from baseline at 2-days following phacoemulsification; (**b**) funnel plot for included meta-analysis studies that provided data on the change in IOP from baseline at 3-days following phacoemulsification; (**c**) funnel plot for included meta-analysis studies that provided data on the change in IOP from baseline at 7-days following phacoemulsification; (**d**) funnel plot for included meta-analysis studies that provided data on the change in IOP from baseline at 30-days following phacoemulsification.

Figure 3 shows the funnel plots for studies that provided data on the change in IOP for days 2, 3, 7, and 30 following phacoemulsification. Included studies for post-operative day 2 were scattered symmetrically on either side of the funnel plot; however, there were only a few data points. The funnel plot appeared to be symmetrical. The funnel plot for post-operative days 3 and 30 had studies scattered slightly asymmetrically, with fewer studies in the bottom left of the plot. Included studies for post-operative day 7 were scattered asymmetrically, with studies missing from the bottom left portion of the plot. As such, this asymmetry implied publication bias at this timepoint. Other potential reasons for asymmetry include difficulty in the interpretation of the funnel plot for a small group of studies, high heterogeneity, and small effect size.

**Figure 4:**
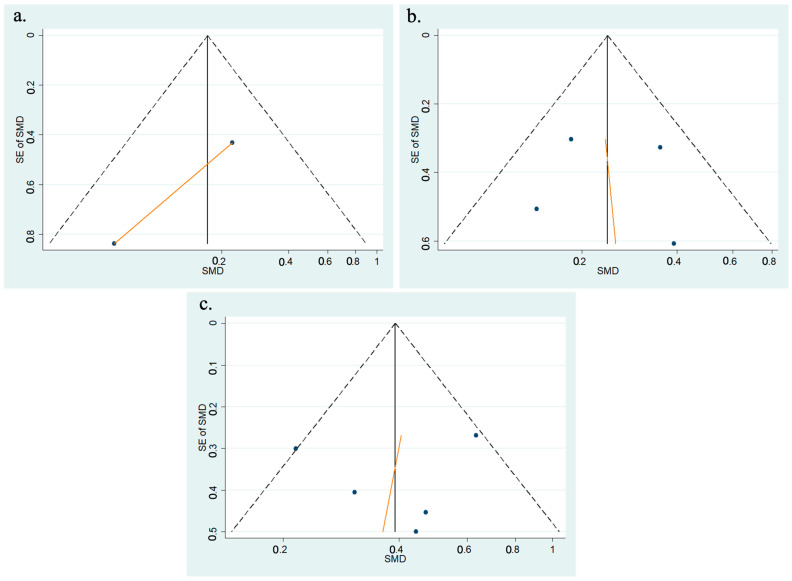
(**a**) Egger’s regression test for included meta-analysis studies that provided data on the change in IOP from baseline at 1 h following phacoemulsification; (**b**) Egger’s regression test for included meta-analysis studies that provided data on the change in IOP from baseline at 4 h following phacoemulsification; (**c**) Egger’s regression test for included meta-analysis studies that provided data on the change in IOP from baseline at 8 h following phacoemulsification.

**Figure 5:**
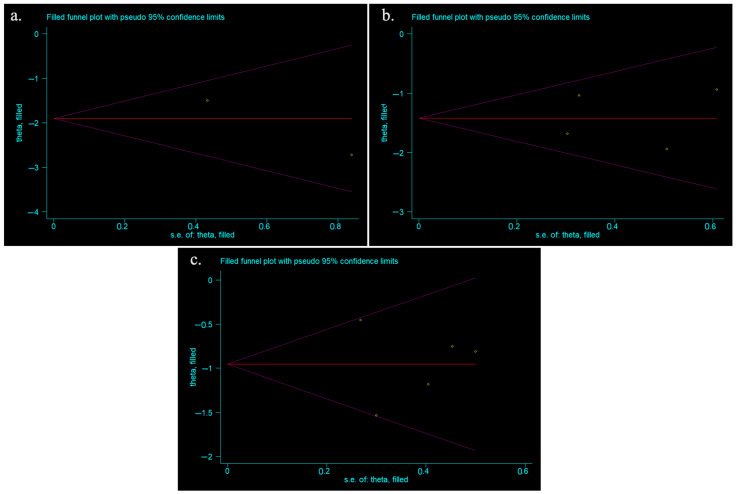
(**a**) Non-parametric Trim and Fill method for included meta-analysis studies that provided data on the change in IOP from baseline at 1 h following phacoemulsification; (**b**) non-parametric Trim and Fill method for included meta-analysis studies that provided data on the change in IOP from baseline at 4 h following phacoemulsification; (**c**) non-parametric Trim and Fill method for included meta-analysis studies that provided data on the change in IOP from baseline at 8 h following phacoemulsification.

Egger’s regression test and trim and fill procedures were performed to further explore potential publication bias for the funnel plots, which appeared asymmetrical at post-operative hours 1, 4, and 8. Funnel plots for hours 1, 4, and 8 include the fitted regression line from the standard Egger’s regression test for small-study effects (Figure 4). However, Egger’s regression test provided weak evidence for the presence of small-study effects. The non-parametric Trim and Fill method of accounting for publication bias in meta-analysis was utilized (Figure 5). For post-operative hours 1, 4, and 8, no “missing” studies were added to the dataset, and, therefore, the variance between studies and evidence of heterogeneity remained unchanged (*p* = 0.0). Therefore, correction for publication bias does not change the overall interpretation of the dataset. 

### 3.5. Effect on IOP

**Figure 6:**
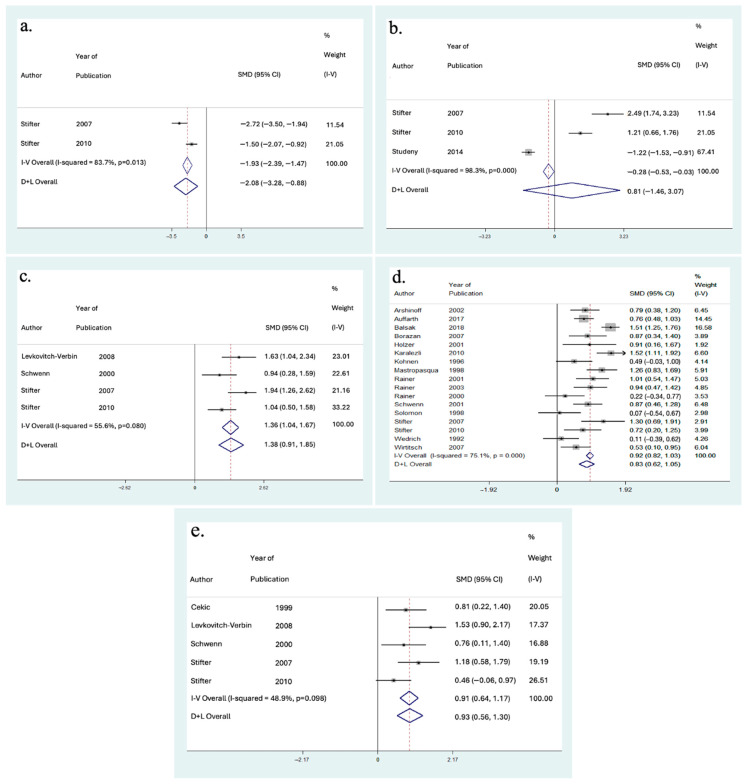
(**a**) Forest plot for included meta-analysis studies that provided data on the change in IOP from baseline at 1 h following phacoemulsification [62,63]; (**b**) Forest plot for included meta-analysis studies that provided data on the change in IOP from baseline at 2 h following phacoemulsification [62,63,64]; (**c**) Forest plot for included meta-analysis studies that provided data on the change in IOP from baseline at 4 h following phacoemulsification [40,57,62,63]; (**d**) Forest plot for included meta-analysis studies that provided data on the change in IOP from baseline at 6 h following phacoemulsification [17,19,20,21,32,36,38,44,50,51,54,58,61,62,63,66,67]; (**e**) Forest plot for included meta-analysis studies that provided data on the change in IOP from baseline at 8 h following phacoemulsification [23,40,57,62,63].

**Figure 7:**
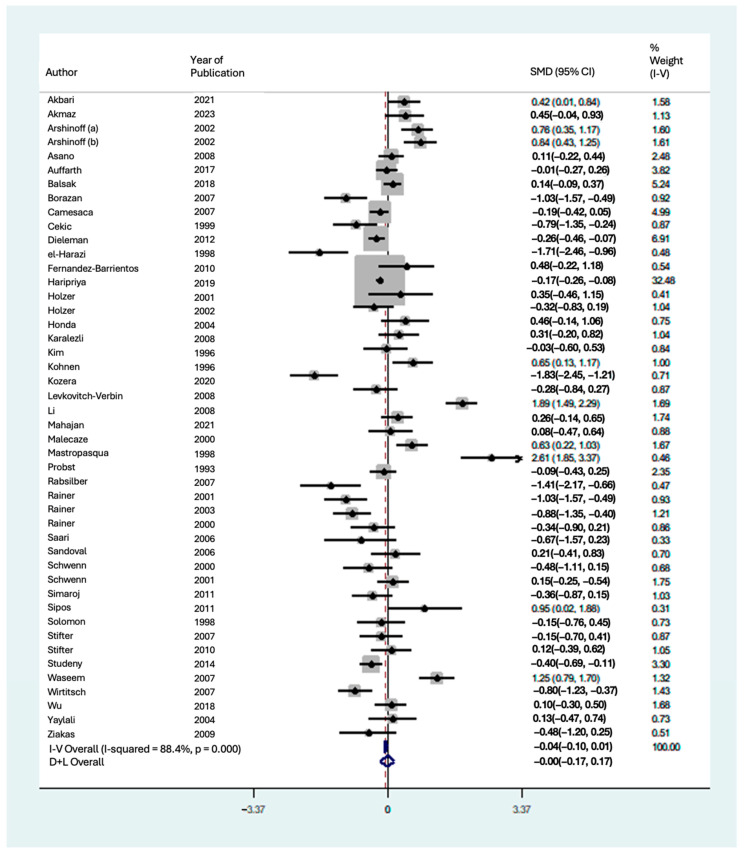
Forest plot for included meta-analysis studies that provided data on the change in IOP from baseline at 1-day following phacoemulsification [15,16,17,18,19,20,21,22,23,27,28,29,31,32,33,34,35,37,38,39,40,41,42,43,44,47,48,50,51,54,55,56,57,58,59,60,61,62,63,64,65,67,68,69,71].

**Figure 8:**
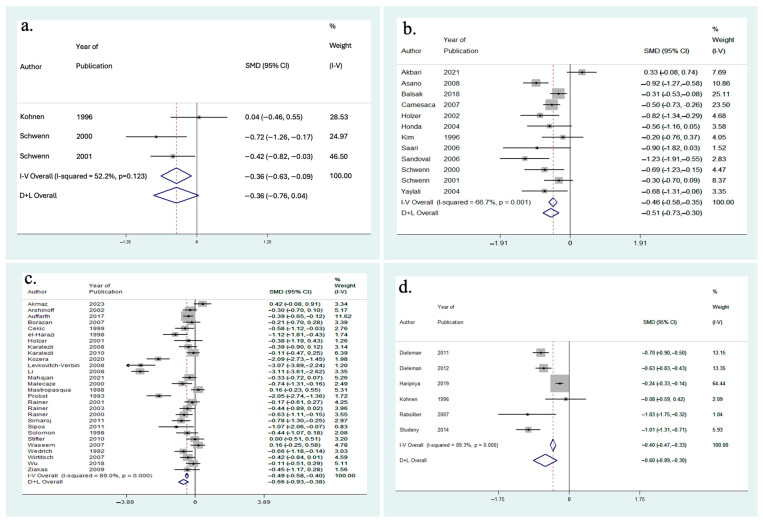
(**a**) Funnel plot for included meta-analysis studies that provided data on the change in IOP from baseline at 2-days following phacoemulsification [38,57,58]; (**b**) Funnel plot for included meta-analysis studies that provided data on the change in IOP from baseline at 3-days following phacoemulsification [15,18,20,22,33,34,37,55,56,57,58,69]; (**c**) Funnel plot for included meta-analysis studies that provided data on the change in IOP from baseline at 7-days following phacoemulsification [16,17,19,21,23,28,32,35,36,39,40,41,42,43,44,47,50,51,54,59,60,61,63,65,66,67,68,71]; (**d**) Funnel plot for included meta-analysis studies that provided data on the change in IOP from baseline at 30-days following phacoemulsification [26,27,31,38,48,64].

A meta-analysis was performed to evaluate the impact of phacoemulsification cataract extraction on IOP in Figure 6 for follow-up at hours 1, 2, 4, 6, and 8, Figure 7 for 1-day follow-up, and Figure 8 for 2-days, 3-days, 7-days, and 30-days follow-up. Significant heterogeneity was found between studies at the 1 h, 2 h, 4 h, 6 h, 1-day, 2-day, and 3-day follow-up periods (I2 = 83.7%, 98.3%, 55.6%, 77.1%, 88.4%, 52.2%, and 66.7% respectively). Thus, a random-effects meta-analysis was performed for these follow-up timepoints using the DerSimonian and Laird method, given the significant heterogeneity among included articles. A few possible explanations for this significant heterogeneity include different study populations, demographics, study location, technique, surgeon’s experience, available facilities to perform surgery, and rates of complications.

**Figure 9:**
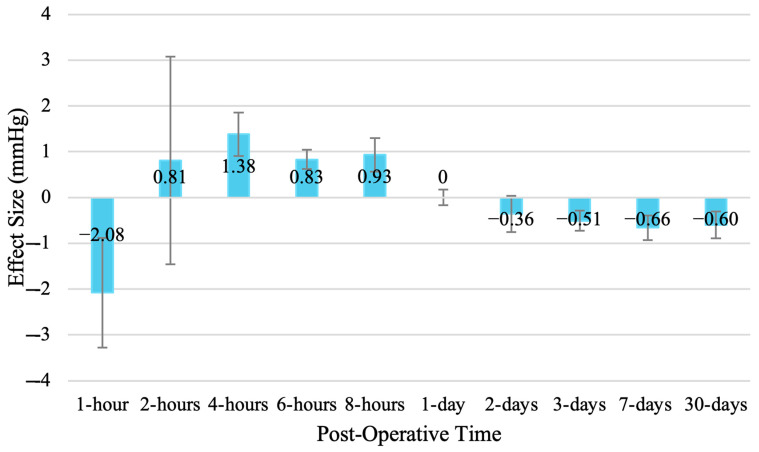
Comparative effect size for the change in IOP from baseline at various timepoints following phacoemulsification.

At 1 h follow-up, the results indicated a significant decrease in IOP from baseline (SMD = −2.08 [95% CI: −3.28 to −0.88]). At 2 h follow-up, the results indicated a non-significant increase in IOP (SMD = +0.81 [95% CI: −1.46 to +3.07]). The results indicated a significant increase in IOP at 4-h follow-up (SMD = +1.38 [95% CI: +0.91 to +1.85]), 6 h follow-up (SMD = +0.83 [95% CI: +0.62 to +1.05]), and 8 h follow-up (SMD = +0.93 [95% CI: +0.56 to +1.30]). At 1-day follow-up, the results indicated there was no change in IOP from baseline (SMD = 0.00 [95% CI: −0.17 to +0.17]). At 2-days follow-up, the results indicated a non-significant decrease in IOP from baseline (SMD = −0.36 [95% CI: −0.76 to +0.04]). The results indicated a significant in IOP from baseline at 3-days follow-up (SMD = −0.51 [95% CI: −0.73 to −0.30]), 7-days follow-up (SMD = −0.66 [95% CI: −0.93 to −0.38]), and 30-days follow-up (SMD = −0.60 [95% CI: −0.89 to −0.30]). Therefore, the results indicated significant decreases in IOP from baseline at 1 h, 3-days, 7-days, and 30-days follow-up (Figure 9). Additionally, there were significant increases in IOP at 4 h, 6 h, and 8 h follow-up. Finally, there was a non-significant increase in IOP at 2 h, no change in IOP at 1-day, and a non-significant decrease in IOP at 2-days follow-up.

### 3.6. Surgical Parameters

Out of the 57 studies included in this meta-analysis, all made use of an OVD in their cataract surgery procedure except for one study [16], which, instead of an OVD, used a balanced salt solution drip for one of its patient samples (Appendix A Table A3). A total of 17 studies used 1.0% Sodium Hyaluronate, 12 used 4.0% Chondroitin sulphate-3.0% sodium hyaluronate, 11 used 1.4% Sodium Hyaluronate, eight studies used 2.0% Hydroxypropyl Methylcellulose, five used 2.3% Sodium Hyaluronate, three studies used 1.2% Sodium Hyaluronate, and two studies used 1.8% Sodium Hyaluronate. Additionally, only one study used each of the following OVDs: 1.5% Sodium Hyaluronate, 1.6% Sodium Hyaluronate, 3.0% Sodium Hyaluronate, 4.0% Chondroitin sulphate-1.65% sodium hyaluronate, 0.1% chitosan, or 2.2% Sodium hyaluronate—1.0% Sodium hyaluronate. Two studies used sodium hyaluronate with an unknown concentration, one study used chondroitin sulfate–sodium hyaluronate with an unknown concentration, and 15 studies did not report which OVD was used. Additionally, all studies included inserting an intraocular lens during the cataract extraction.

Table A3 found in Appendix A describes the different phacoemulsification techniques used in each study as described by the respective authors. A total of 28 studies reported initial incision size, which ranged from 2.2 to 6.0 mm. Of the 20 studies that reported incision location, 14 used a temporal-placed incision as opposed to the six that used a superior-located incision. Additionally, of the 22 studies that reported incision placement, 17 used a clear corneal while only three used a corneoscleral incision, and two used a limbal incision. A total of 10 studies reported capsulorrhexis size, which ranged from 4.0 to 6.0 mm. A total of 12 studies reported their procedure to follow what was deemed as a traditional or standard phacoemulsification technique. Four studies did not report the surgical technique that was performed.

In terms of the method used to measure IOP in each study, 44 studies reported using Goldmann Applanation Tonometry, four used Non-Contact Tonometry, one used Pneumotonometry, and eight did not report which method was used (Appendix A Table A3). A total of 13 studies measured clear corneal thickness (CCT) through pachymetry, while 44 studies did not (Appendix A Table A3). Out of those studies that did measure CCT, none found a statistically significant difference, and only one study [70] corrected for the effect CCT had on IOP measurements. No studies reported correcting IOP based on astigmatism. Out of the 57 studies included in the meta-analysis, only one publication [70] reported the exact time of day when IOP was measured, which recorded baseline IOP between 8 a.m. and 4 p.m. No studies reported on the patient’s position during IOP measurement.

### 3.7. Medications Administered

Out of the 57 included studies, 3 did not report which medications were used in their protocol (Appendix A Table A4). In terms of pre-operative medications, 12 studies administered a non-steroidal anti-inflammatory (NSAID) and, out of those, 11 were diclofenac and one was ketorolac. Five studies administered an antibiotic, with four being a fluoroquinolone and one being an aminoglycoside antibiotic. Additionally, one study administered acetazolamide, one an unspecified anti-glaucoma medication, one an unspecified antibiotic eye drops, and 31 studies reported administering no post-operative medications. Regarding intra-operative medications, five studies administered an antibiotic, with three being a fluoroquinolone, one being a cephalosporin, and one being an aminoglycoside antibiotic. Additionally, five studies administered carbachol, four administered triamcinolone acetonide, one study gave trypan blue, one administered epinephrine, and one gave a fixed dorzolamide–timolol combination. A total of 38 studies did not report medications intraoperatively. Finally, for post-operative medications, 35 studies administered an antibiotic, of which 14 were aminoglycoside, eight fluoroquinolone, five amphenicol, two quinolone, one polypeptide, and one fusidane, and four were unspecified. A total of 27 studies administered a corticosteroid, of those 16 were dexamethasone, six betamethasone, two loteprednol, two triamcinolone acetonide, and one was difluprednate. A total of 20 studies administered a glucocorticoid, of those 18 were prednisolone acetate, one rimexolone, and one was fluorometholone. Additionally, four studies administered an unspecified steroid. A total of 15 studies administered an NSAID, of which 10 used diclofenac, four used ketorolac, and one used indomethacin. Three studies used a dorzolamide–timolol fixed combo, three gave brimonidine, two acetazolamide, two timolol, one dorzolamide, one latanoprost, one brinzolamide, one apraclonidine, one gave an unspecified topical beta-blocker, one study gave an unspecified glaucoma medication, and one study administered both a low-viscosity and high-viscosity tear replacement. Finally, one study administered physostigmine, one gave carbachol, and five studies did not give any post-operative medications.

## 4. Discussion

A systematic review and a meta-analysis were conducted to determine the current status of evidence that may help guide best practice on the optimal time following phacoemulsification cataract surgery to measure post-operative IOP. Various database searches of randomized clinical trial studies were conducted to measure the change in post-operative IOP compared to pre-operative baseline measurements. The use of OVD, the surgical procedure employed during cataract extraction, and the medications administered were also recorded and analyzed.

The findings from the included articles consistently showed a significant drop in IOP at the 1 h follow-up timepoint. While IOP rose slightly but non-significantly at 2 h, it subsequently increased significantly at 4, 6, and 8 h, reaching its peak elevation during this period. At the 1-day follow-up, IOP returned to baseline levels and then declined non-significantly at the 2-days timepoint. A significant decrease in IOP from baseline was observed for the follow-up timepoints of 3-days, 7-days, and 30-days, marking the first measurements outside the initial 48 h postoperative window. The majority of studies measured IOP using the gold standard of Goldmann Applanation Tonometry [72].

These results identified 4 to 8 h following surgery as the optimal time to identify a post-operative IOP spike. The findings also suggested that measuring the IOP sooner than 2 h may be misleading as IOPs were found to be lower than baseline, likely related to surgical techniques to manage wound closure following aspiration of OVD. The results indicated that a continuous decrease in IOP at the 3-days, 7-days, and 30-days is expected and consistent with evidence that retained OVD is unlikely to be causative after 2–3 days [8]. This would suggest that other causes, such as steroid response, would be a more likely consideration, especially at the 30-day mark [73].

The results also demonstrated the significant variability in peri-operative medications used at the time of cataract surgery. A total of 54 studies reported the medications administered at pre-operative, intra-operative, and post-operative stages (Appendix A Table A4). The most common pre-operative medications were either an antibiotic or an NSAID. Intra-operatively, the majority of studies did not report the administration of any medications, but, for those that did, the most common medication given was either an antibiotic, carbachol, and/or a steroid. Post-operatively, the majority of studies used a combination of either an antibiotic, steroid, and/or NSAID. These medication combinations have been shown in some studies to have a small effect on IOP in the immediate days following cataract surgery [74]. Ten studies reported the use of an IOP-lowering medication, which included carbonic anhydrase inhibitors, beta-blockers, prostaglandin analogs, and alpha agonists [75]. These medications were used either prophylactically to prevent IOP elevations, to lower IOP in patients who were actively experiencing a dangerous spike, or as routine treatment for glaucoma patients. Despite these medications having peak effects between 2 and 8 h post-administration, they did not seem to prevent the IOP spikes noted from 4 to 8 h later [75].

Six of the studies included patients with a known diagnosis of glaucoma or pseudoexfoliation. Only one study measured IOP at the 4 h and 8 h follow-ups, at which time they noted a significant elevation in IOP from baseline at both timepoints [40]. Additionally, all six publications measured IOP at the 1-day follow-up, at which three saw a slight elevation in IOP while three saw a slight decrease in IOP. These results suggested that perhaps further studies should be performed to more fully understand the risk of IOP spikes in patients with glaucoma and pseudoexfoliation undergoing standalone cataract surgery.

The following limitations should be considered in understanding the context of the results. First, there was some variability in the quality scoring of the included studies. This can be seen in the risk of bias assessment, as a few studies provided unclear information surrounding blinding (Appendix A Table A2). However, as the current literature only offered a small number of relevant publications, all were included, irrespective of their quality score. A second limitation was the asymmetry of a few funnel plots at various post-operative follow-up timepoints. However, after further investigation, Egger’s regression test provided weak evidence for the presence of small-study effects. Additionally, the non-parametric Trim and Fill method indicated correction for publication bias did not change the overall interpretation of the dataset. Therefore, the asymmetry of the funnel plots was most likely not due to publication bias. Asymmetry could have been caused by several other reasons, including difficulty in the interpretation of the funnel plot for a small group of studies, high heterogeneity, and small effect sizes. A third limitation was that only one study corrected for the effect CCT values had on IOP measurements, and no studies corrected for the effect potential astigmatism had on IOP measurements. Additionally, no studies reported patient position during IOP measurement. This is important to note as CCT, astigmatism, and patient position can potentially affect IOP measurements [76,77]. Finally, there was significant heterogeneity at all post-operative timepoints except for 8 h following phacoemulsification cataract surgery. A few possible explanations for this include different study populations, demographics, study location, technique, surgeon’s experience, available facilities to perform surgery, and rates of complications.

## 5. Conclusions

Existing relevant studies consistently indicated a noticeable rise in IOP from baseline 4, 6, and 8 h following phacoemulsification cataract surgery. If measurements were made sooner, they risked missing these spikes, which could result in both acute and longer-term negative patient outcomes [12]. Patients’ perceptions of outcomes and expectations are increasingly becoming considered an important element in healthcare decision-making [78]. Advancements in cataract surgery have elevated patient expectations in terms of how quickly they achieve an improved visual acuity as well as their overall surgical experience. Continuous practice assessment to determine factors that optimize patient-reported outcomes and experiences in the context of evidence-based quality care may be the key to best practice recommendations.

## Data Availability

The original contributions presented in the study are included in the article, further inquiries can be directed to the corresponding authors.

## Appendix A

**Table A1:** Study characteristics of included studies.

Author	Year	Study Design	Study Location	Total Number of Participants, N	Total Number of Eyes, N	Age, Mean (SD); Range; N (%), etc.	Male Eyes, N (%)	Disease Population of Interest
Akbari et al. [15]	2021	Randomized Clinical Trial	Iran	88	88	71.6 (7.8)	41 (46.6)	Cataract with PEX
Akmaz et al. [16]	2023	Randomized Clinical Trial	Turkey	65	65	66.6 (8.8)	34 (52.3)	Cataract only
Arshinoff et al. [17]	2002	Randomized Clinical Trial	Canada	99	198	71.7 (8.6)	96 (48.5)	Cataract only
Asano et al. [18]	2008	Randomized Clinical Trial	Japan	142	142	66.2 (5.5)	63 (44.4)	Cataract only
Auffarth et al. [19]	2017	Randomized Clinical Trial	Multicenter (Europe)	220	220	72.2 (7.6)	84 (38.2)	Cataract only
Balsak et al. [20]	2018	Randomized Clinical Trial	Turkey	257	277	65.9 (13.9)	142 (51.2)	Cataract only
Borazan et al. [21]	2007	Randomized Clinical Trial	Turkey	185	185	67.1 (8.3)	107 (57.8)	Cataract only
Camesaca et al. [22]	2007	Randomized Clinical Trial	Italy	143	286	73.7 (8.9)	53 (18.5)	Cataract only
Cekic et al. [23]	1999	Randomized Clinical Trial	Turkey	51	51	55.7 (-)	30 (58.8)	Cataract only
Cekic et al. [24]	1999	Randomized Clinical Trial	Turkey	58	58	57.3 (31–78)	35 (60.3)	Cataract only
Chang et al. [25]	2017	Randomized Clinical Trial	Sweden	43	43	69.5 (8.3)	-	Cataract only
Dieleman et al. [26]	2011	Randomized Clinical Trial	Netherlands	400	400	70.8 (41–91)	158 (39.5)	Cataract only
Dieleman et al. [27]	2012	Randomized Clinical Trial	Netherlands	400	400	70.8 (41–93)	158 (39.5)	Cataract only
el-Harazi et al. [28]	1998	Randomized Clinical Trial	United States	58	58	69.8 (11.0)	30 (51.7)	Cataract only
Fernandez-Barrientos et al. [29]	2010	Randomized Clinical Trial	Spain	16	16	76.7 (5.8)	9 (56.3)	Cataract with Glaucoma
Gungor et al. [30]	2014	Randomized Clinical Trial	Turkey	60	60	70.4 (9.9)	24 (40.0)	Cataract only
Haripriya et al. [31]	2019	Randomized Clinical Trial	India	1406	1406	61.3 (7.4)	717 (51.0)	Cataract only and, Cataract with PEX
Holzer et al. [32]	2001	Randomized Clinical Trial	United States	81	81	71.2 (7.6)	-	Cataract only
Holzer et al. [33]	2002	Randomized Clinical Trial	Germany	60	60	67.9 (*p* = 0.62)	21 (35.0)	Cataract only
Honda et al. [34]	2004	Randomized Clinical Trial	Japan	44	44	61.0 (9.5)	23 (52.3)	Cataract only
Karalezli et al. [35]	2008	Randomized Clinical Trial	Turkey	60	60	66.0 (7.1)	27 (45)	Cataract only
Karalezli et al. [36]	2010	Randomized Clinical Trial	Turkey	120	120	66.6 (8.7)	68 (56.7)	Cataract only
Kim et al. [37]	1996	Randomized Clinical Trial	Korea	24	24	63.0 (5.5)	9 (37.5)	Cataract only
Kohnen et al. [38]	1996	Randomized Clinical Trial	Germany	60	60	73.2 (9.1)	-	Cataract only
Kozera et al. [39]	2020	Randomized Clinical Trial	Poland	36	36	71.8 (6.7)	8 (22.2)	Cataract with Glaucoma
Levkovitch-Verbin et al. [40]	2008	Randomized Clinical Trial	Israel	122	122	75.0 (3.8)	53 (43.4)	Cataract only, Cataract with Glaucoma, and Cataract with PEX
Li et al. [41]	2008	Randomized Clinical Trial	China	140	140	72.4 (9.5)	63 (45.0)	Cataract only
Mahajan et al. [42]	2021	Randomized Clinical Trial	India	150	150	64.4 (9.4)	72 (48.0)	Cataract only
Malecaze et al. [43]	2000	Randomized Clinical Trial	France	50	50	75.0 (70–79)	21 (42.0)	Cataract only
Mastropasqua et al. [44]	1998	Randomized Clinical Trial	Italy	100	100	68.2 (11.9)	55 (55.0)	Cataract only
Paganelli et al. [45]	2004	Randomized Clinical Trial	Brazil	100	100	65.2 (7.9)	48 (48.0)	Cataract only
Paganelli et al. [46]	2009	Randomized Clinical Trial	Brazil	135	135	69.2 (8.0)	54 (40.0)	Cataract only
Probst et al. [47]	1993	Randomized Clinical Trial	Canada	49	50	67.6 (12.4)	28 (56.0)	Cataract only
Rabsilber et al. [48]	2007	Randomized Clinical Trial	Germany	17	34	70.1 (9.7)	12 (35.3)	Cataract only
Rainer et al. [49]	2000	Randomized Clinical Trial	Austria	25	50	75.7 (10.5)	20 (40.0)	Cataract only
Rainer et al. [50]	2000	Randomized Clinical Trial	Austria	35	70	75.5 (9.1)	28 (40.0)	Cataract only
Rainer et al. [51]	2001	Randomized Clinical Trial	Austria	40	80	75.9 (9.3)	26 (32.5)	Cataract only
Rainer et al. [52]	2001	Randomized Clinical Trial	Austria	40	80	69.9 (12.1)	30 (37.5)	Cataract only
Rainer et al. [53]	2001	Randomized Clinical Trial	Austria	30	60	64.8 (15.2)	12 (20)	Cataract only
Rainer et al. [54]	2003	Randomized Clinical Trial	Austria	38	76	75.6 (10.9)	10 (13.2)	Cataract only
Saari et al. [55]	2006	Randomized Clinical Trial	Finland	20	20	72.0 (6.3)	8 (40.0)	Cataract only
Sandoval et al. [56]	2006	Randomized Clinical Trial	United States	40	40	71.5 (8.4)	18 (45.0)	Cataract only
Schwenn et al. [57]	2000	Randomized Clinical Trial	Germany	48	48	-	-	Cataract only
Schwenn et al. [58]	2001	Randomized Clinical Trial	Germany	100	100	71.2 (12.9)	66 (66.0)	Cataract only
Simaroj et al. [59]	2011	Randomized Clinical Trial	Thailand	60	60	66.1 (8.1)	24 (40.0)	Cataract only
Sipos et al. [60]	2011	Randomized Clinical Trial	Austria	30	60	75.2 (8.3)	22 (36.7)	Cataract only
Solomon et al. [61]	1998	Randomized Clinical Trial	United States	41	41	66.9 (11.5)	26 (63.4)	Cataract only
Stifter et al. [62]	2007	Randomized Clinical Trial	Austria	50	100	77.0 (8.4)	38 (38.0)	Cataract only
Stifter et al. [63]	2010	Randomized Clinical Trial	Austria	30	60	79.9 (6.2)	26 (43.0)	Cataract only
Studeny et al. [64]	2014	Randomized Clinical Trial	Czech Republic	96	192	70.2 (8.3)	70 (36.5)	Cataract only
Waseem et al. [65]	2007	Randomized Clinical Trial	Pakistan	91	91	-	51 (56.0)	Cataract only
Wedrich et al. [66]	1992	Randomized Clinical Trial	Austria	90	90	76.5 (7.2)	11 (12.2)	Cataract only
Wirtitsch et al. [67]	2007	Randomized Clinical Trial	Austria	44	88	76.0 (8.5)	22 (25.0)	Cataract only
Wu et al. [68]	2018	Randomized Clinical Trial	China	81	99	70.0 (10.0)	21 (21.2)	Cataract only
Yaylali et al. [69]	2004	Randomized Clinical Trial	Turkey	48	48	61.9 (12.2)	26 (54.2)	Cataract only
Zebardast et al. [70]	2020	Randomized Clinical Trial	Multicentre (Canada, USA, Germany, Italy, Mexico, Philippines, Poland, Spain, UK)	187	187	71.2 (7.6)	82 (43.8)	Cataract with Glaucoma
Ziakas et al. [71]	2009	Randomized Clinical Trial	Greece	15	30	72.6 (5.7)	16 (53.3)	Cataract only

**Table A2:** Results of risk of bias assessment for studies included.

First Author	Year	1. Was the Allocation Sequence Adequately Generated?	2. Was the Allocation Adequately Concealed?	3a. Were Patients Blinded?	3b. Were Healthcare Providers Blinded?	3c. Were Data Collectors Blinded?	3d. Were Outcome Assessors Blinded?	3e. Were Data Analysts Blinded?	4. Was Loss to Follow-Up (Missing Outcome Data) Infrequent?	5. Are Reports of the Study Free of Selective Outcome Reporting?	6. Was the Study Apparently Free of Other Problems That Could Put It at a Risk of Bias?
Akbari et al. [15]	2021	Definitely yes (low risk of bias)	Probably no	Definitely yes (low risk of bias)	Definitely no (high risk of bias)	Definitely no (high risk of bias)	Definitely no (high risk of bias)	Definitely no (high risk of bias)	Probably no	Definitely yes (low risk of bias)	Definitely yes (low risk of bias)
Akmaz et al. [16]	2023	Definitely yes (low risk of bias)	Probably no	Probably no	Definitely no (high risk of bias)	Probably no	Probably no	Probably no	Definitely yes (low risk of bias)	Definitely yes (low risk of bias)	Definitely yes (low risk of bias)
Arshinoff et al. [17]	2002	Definitely yes (low risk of bias)	Probably no	Definitely yes (low risk of bias)	Probably no	Definitely yes (low risk of bias)	Definitely yes (low risk of bias)	Definitely yes (low risk of bias)	Definitely yes (low risk of bias)	Definitely yes (low risk of bias)	Definitely no (high risk of bias)
Asano et al. [18]	2008	Probably no	Definitely yes (low risk of bias)	Definitely yes (low risk of bias)	Definitely yes (low risk of bias)	Definitely yes (low risk of bias)	Definitely yes (low risk of bias)	Definitely yes (low risk of bias)	Definitely yes (low risk of bias)	Definitely yes (low risk of bias)	Probably yes
Auffarth et al. [19]	2017	Definitely yes (low risk of bias)	Definitely yes (low risk of bias)	Definitely yes (low risk of bias)	Definitely no (high risk of bias)	Definitely yes (low risk of bias)	Definitely yes (low risk of bias)	Probably yes	Definitely yes (low risk of bias)	Definitely yes (low risk of bias)	Probably yes
Balsak et al. [20]	2018	Probably no	Probably no	Definitely yes (low risk of bias)	Definitely yes (low risk of bias)	Definitely yes (low risk of bias)	Definitely yes (low risk of bias)	Definitely yes (low risk of bias)	Probably yes	Definitely yes (low risk of bias)	Definitely yes (low risk of bias)
Borazan et al. [21]	2007	Probably no	Probably no	Definitely yes (low risk of bias)	Definitely yes (low risk of bias)	Definitely yes (low risk of bias)	Definitely yes (low risk of bias)	Definitely yes (low risk of bias)	Probably yes	Definitely yes (low risk of bias)	Probably yes
Camesaca et al. [22]	2007	Probably no	Probably no	Probably no	Probably no	Probably no	Probably no	Probably no	Probably yes	Definitely yes (low risk of bias)	Probably yes
Cekic et al. [23]	1999	Probably no	Probably no	Probably no	Probably no	Probably no	Probably no	Probably no	Definitely no (high risk of bias)	Probably yes	Probably yes
Cekic et al. [24]	1999	Probably no	Probably no	Probably no	Definitely no (high risk of bias)	Probably no	Probably no	Probably no	Probably yes	Definitely yes (low risk of bias)	Probably yes
Chang et al. [25]	2017	Definitely yes (low risk of bias)	Probably no	Definitely yes (low risk of bias)	Definitely no (high risk of bias)	Definitely no (high risk of bias)	Definitely no (high risk of bias)	Definitely no (high risk of bias)	Definitely yes (low risk of bias)	Probably yes	Definitely yes (low risk of bias)
Dieleman et al. [26]	2011	Definitely yes (low risk of bias)	Definitely yes (low risk of bias)	Definitely no (high risk of bias)	Definitely no (high risk of bias)	Definitely no (high risk of bias)	Definitely no (high risk of bias)	Definitely no (high risk of bias)	Definitely yes (low risk of bias)	Definitely yes (low risk of bias)	Definitely yes (low risk of bias)
Dieleman et al. [27]	2012	Probably no	Probably no	Definitely no (high risk of bias)	Definitely no (high risk of bias)	Definitely no (high risk of bias)	Definitely no (high risk of bias)	Definitely no (high risk of bias)	Definitely yes (low risk of bias)	Definitely no (high risk of bias)	Definitely yes (low risk of bias)
el-Harazi et al. [28]	1998	Probably no	Definitely yes (low risk of bias)	Definitely yes (low risk of bias)	Definitely yes (low risk of bias)	Definitely yes (low risk of bias)	Definitely yes (low risk of bias)	Definitely yes (low risk of bias)	Definitely yes (low risk of bias)	Definitely yes (low risk of bias)	Probably yes
Fernandez-Barrientos et al. [29]	2010	Definitely yes (low risk of bias)	Probably no	Definitely yes (low risk of bias)	Definitely no (high risk of bias)	Definitely yes (low risk of bias)	Definitely yes (low risk of bias)	Probably yes	Definitely yes (low risk of bias)	Definitely yes (low risk of bias)	Definitely no (high risk of bias)
Gungor et al. [30]	2014	Probably no	Probably no	Definitely no (high risk of bias)	Definitely no (high risk of bias)	Definitely no (high risk of bias)	Definitely no (high risk of bias)	Definitely no (high risk of bias)	Definitely yes (low risk of bias)	Definitely yes (low risk of bias)	Probably yes
Haripriya et al. [31]	2019	Definitely yes (low risk of bias)	Definitely yes (low risk of bias)	Definitely yes (low risk of bias)	Definitely no (high risk of bias)	Probably no	Probably no	Probably no	Definitely no (high risk of bias)	Definitely yes (low risk of bias)	Probably yes
Holzer et al. [32]	2001	Probably no	Probably no	Definitely yes (low risk of bias)	Definitely no (high risk of bias)	Definitely yes (low risk of bias)	Definitely yes (low risk of bias)	Definitely yes (low risk of bias)	Probably yes	Definitely yes (low risk of bias)	Definitely yes (low risk of bias)
Holzer et al. [33]	2002	Probably no	Definitely yes (low risk of bias)	Definitely yes (low risk of bias)	Definitely yes (low risk of bias)	Definitely yes (low risk of bias)	Definitely yes (low risk of bias)	Definitely yes (low risk of bias)	Probably yes	Definitely yes (low risk of bias)	Definitely no (high risk of bias)
Honda et al. [34]	2004	Probably no	Probably no	Definitely no (high risk of bias)	Definitely no (high risk of bias)	Definitely no (high risk of bias)	Definitely no (high risk of bias)	Definitely no (high risk of bias)	Definitely yes (low risk of bias)	Definitely yes (low risk of bias)	Probably yes
Karalezli et al. [35]	2008	Definitely yes (low risk of bias)	Definitely yes (low risk of bias)	Definitely no (high risk of bias)	Definitely no (high risk of bias)	Definitely no (high risk of bias)	Definitely no (high risk of bias)	Definitely no (high risk of bias)	Definitely yes (low risk of bias)	Probably yes	Probably yes
Karalezli et al. [36]	2010	Definitely yes (low risk of bias)	Probably no	Probably no	Definitely no (high risk of bias)	Definitely no (high risk of bias)	Definitely no (high risk of bias)	Definitely no (high risk of bias)	Definitely yes (low risk of bias)	Definitely yes (low risk of bias)	Probably yes
Kim et al. [37]	1996	Probably no	Probably no	Probably no	Definitely no (high risk of bias)	Probably no	Probably no	Probably no	Definitely yes (low risk of bias)	Probably yes	Probably yes
Kohnen et al. [38]	1996	Probably no	Probably no	Probably no	Probably no	Probably no	Probably no	Probably no	Probably yes	Definitely yes (low risk of bias)	Definitely no (high risk of bias)
Kozera et al. [39]	2020	Definitely yes (low risk of bias)	Definitely yes (low risk of bias)	Probably no	Definitely no (high risk of bias)	Probably no	Probably no	Probably no	Definitely no (high risk of bias)	Definitely yes (low risk of bias)	Definitely yes (low risk of bias)
Levkovitch-Verbin et al. [40]	2008	Definitely yes (low risk of bias)	Definitely yes (low risk of bias)	Definitely yes (low risk of bias)	Definitely yes (low risk of bias)	Definitely yes (low risk of bias)	Definitely yes (low risk of bias)	Definitely yes (low risk of bias)	Definitely yes (low risk of bias)	Definitely yes (low risk of bias)	Probably yes
Li et al. [41]	2008	Definitely yes (low risk of bias)	Probably no	Definitely yes (low risk of bias)	Definitely no (high risk of bias)	Definitely yes (low risk of bias)	Definitely yes (low risk of bias)	Definitely yes (low risk of bias)	Probably yes	Definitely yes (low risk of bias)	Definitely no (high risk of bias)
Mahajan et al. [42]	2021	Definitely yes (low risk of bias)	Definitely yes (low risk of bias)	Probably no	Probably no	Probably no	Probably no	Probably no	Definitely yes (low risk of bias)	Definitely yes (low risk of bias)	Definitely yes (low risk of bias)
Malecaze et al. [43]	2000	Definitely yes (low risk of bias)	Definitely yes (low risk of bias)	Definitely yes (low risk of bias)	Definitely yes (low risk of bias)	Definitely yes (low risk of bias)	Definitely yes (low risk of bias)	Definitely yes (low risk of bias)	Definitely yes (low risk of bias)	Definitely yes (low risk of bias)	Definitely yes (low risk of bias)
Mastropasqua et al. [44]	1998	Probably no	Probably no	Probably no	Probably no	Probably no	Probably no	Probably no	Definitely yes (low risk of bias)	Definitely yes (low risk of bias)	Probably yes
Paganelli et al. [45]	2004	Definitely yes (low risk of bias)	Probably no	Definitely yes (low risk of bias)	Definitely yes (low risk of bias)	Definitely yes (low risk of bias)	Definitely yes (low risk of bias)	Definitely yes (low risk of bias)	Definitely yes (low risk of bias)	Definitely yes (low risk of bias)	Definitely yes (low risk of bias)
Paganelli et al. [46]	2009	Definitely yes (low risk of bias)	Definitely yes (low risk of bias)	Definitely yes (low risk of bias)	Definitely yes (low risk of bias)	Definitely yes (low risk of bias)	Definitely yes (low risk of bias)	Definitely yes (low risk of bias)	Definitely yes (low risk of bias)	Definitely yes (low risk of bias)	Probably yes
Probst et al. [47]	1993	Probably no	Probably no	Probably no	Definitely no (high risk of bias)	Definitely no (high risk of bias)	Definitely no (high risk of bias)	Definitely no (high risk of bias)	Probably yes	Definitely yes (low risk of bias)	Definitely yes (low risk of bias)
Rabsilber et al. [48]	2007	Definitely yes (low risk of bias)	Definitely yes (low risk of bias)	Probably no	Probably no	Probably no	Probably no	Probably no	Probably yes	Definitely yes (low risk of bias)	Definitely yes (low risk of bias)
Rainer et al. [49]	2000	Probably no	Probably no	Definitely yes (low risk of bias)	Definitely yes (low risk of bias)	Definitely yes (low risk of bias)	Definitely yes (low risk of bias)	Definitely yes (low risk of bias)	Definitely yes (low risk of bias)	Definitely yes (low risk of bias)	Definitely yes (low risk of bias)
Rainer et al. [50]	2000	Probably no	Probably no	Definitely yes (low risk of bias)	Definitely no (high risk of bias)	Definitely yes (low risk of bias)	Definitely yes (low risk of bias)	Definitely yes (low risk of bias)	Definitely yes (low risk of bias)	Definitely yes (low risk of bias)	Definitely yes (low risk of bias)
Rainer et al. [51]	2001	Probably no	Probably no	Definitely yes (low risk of bias)	Definitely no (high risk of bias)	Definitely yes (low risk of bias)	Definitely yes (low risk of bias)	Definitely yes (low risk of bias)	Definitely yes (low risk of bias)	Definitely yes (low risk of bias)	Definitely yes (low risk of bias)
Rainer et al. [52]	2001	Probably no	Probably no	Definitely yes (low risk of bias)	Definitely yes (low risk of bias)	Definitely yes (low risk of bias)	Definitely yes (low risk of bias)	Definitely yes (low risk of bias)	Definitely yes (low risk of bias)	Definitely yes (low risk of bias)	Definitely yes (low risk of bias)
Rainer et al. [53]	2001	Probably no	Probably no	Definitely yes (low risk of bias)	Definitely yes (low risk of bias)	Definitely yes (low risk of bias)	Definitely yes (low risk of bias)	Definitely yes (low risk of bias)	Definitely yes (low risk of bias)	Definitely yes (low risk of bias)	Definitely yes (low risk of bias)
Rainer et al. [54]	2003	Probably no	Probably no	Definitely yes (low risk of bias)	Definitely yes (low risk of bias)	Definitely yes (low risk of bias)	Definitely yes (low risk of bias)	Definitely yes (low risk of bias)	Definitely yes (low risk of bias)	Definitely yes (low risk of bias)	Definitely yes (low risk of bias)
Saari et al. [55]	2006	Probably no	Probably no	Probably no	Definitely yes (low risk of bias)	Definitely yes (low risk of bias)	Definitely yes (low risk of bias)	Definitely yes (low risk of bias)	Definitely yes (low risk of bias)	Definitely yes (low risk of bias)	Probably yes
Sandoval et al. [56]	2006	Probably no	Definitely yes (low risk of bias)	Definitely yes (low risk of bias)	Definitely yes (low risk of bias)	Definitely yes (low risk of bias)	Definitely yes (low risk of bias)	Definitely yes (low risk of bias)	Definitely yes (low risk of bias)	Definitely yes (low risk of bias)	Definitely no (high risk of bias)
Schwenn et al. [57]	2000	Definitely yes (low risk of bias)	Probably no	Definitely yes (low risk of bias)	Definitely no (high risk of bias)	Definitely yes (low risk of bias)	Definitely yes (low risk of bias)	Definitely yes (low risk of bias)	Probably no	Definitely yes (low risk of bias)	Definitely yes (low risk of bias)
Schwenn et al. [58]	2001	Probably no	Probably no	Probably no	Definitely no (high risk of bias)	Probably no	Probably no	Probably no	Probably no	Definitely yes (low risk of bias)	Definitely yes (low risk of bias)
Simaroj et al. [59]	2011	Probably no	Probably no	Definitely no (high risk of bias)	Definitely no (high risk of bias)	Probably no	Probably no	Probably no	Definitely yes (low risk of bias)	Definitely yes (low risk of bias)	Definitely yes (low risk of bias)
Sipos et al. [60]	2011	Probably no	Probably no	Probably no	Definitely yes (low risk of bias)	Definitely yes (low risk of bias)	Definitely yes (low risk of bias)	Definitely yes (low risk of bias)	Definitely yes (low risk of bias)	Definitely yes (low risk of bias)	Definitely yes (low risk of bias)
Solomon et al. [61]	1998	Probably no	Definitely yes (low risk of bias)	Definitely yes (low risk of bias)	Definitely yes (low risk of bias)	Definitely yes (low risk of bias)	Definitely yes (low risk of bias)	Definitely yes (low risk of bias)	Definitely yes (low risk of bias)	Definitely yes (low risk of bias)	Definitely no (high risk of bias)
Stifter et al. [62]	2007	Probably no	Probably no	Definitely yes (low risk of bias)	Definitely no (high risk of bias)	Definitely yes (low risk of bias)	Definitely yes (low risk of bias)	Definitely yes (low risk of bias)	Definitely yes (low risk of bias)	Definitely yes (low risk of bias)	Definitely yes (low risk of bias)
Stifter et al. [63]	2010	Definitely yes (low risk of bias)	Definitely yes (low risk of bias)	Definitely yes (low risk of bias)	Definitely no (high risk of bias)	Definitely yes (low risk of bias)	Definitely yes (low risk of bias)	Definitely yes (low risk of bias)	Definitely yes (low risk of bias)	Definitely yes (low risk of bias)	Definitely yes (low risk of bias)
Studeny et al. [64]	2014	Definitely yes (low risk of bias)	Probably no	Definitely yes (low risk of bias)	Definitely no (high risk of bias)	Definitely yes (low risk of bias)	Definitely yes (low risk of bias)	Definitely yes (low risk of bias)	Definitely yes (low risk of bias)	Definitely yes (low risk of bias)	Definitely yes (low risk of bias)
Waseem et al. [65]	2007	Probably no	Probably no	Definitely yes (low risk of bias)	Definitely no (high risk of bias)	Definitely yes (low risk of bias)	Definitely yes (low risk of bias)	Definitely yes (low risk of bias)	Probably yes	Definitely yes (low risk of bias)	Definitely yes (low risk of bias)
Wedrich et al. [66]	1992	Probably no	Probably no	Probably no	Probably no	Probably no	Probably no	Probably no	Probably yes	Definitely yes (low risk of bias)	Probably yes
Wirtitsch et al. [67]	2007	Probably no	Probably no	Definitely yes (low risk of bias)	Definitely no (high risk of bias)	Definitely yes (low risk of bias)	Definitely yes (low risk of bias)	Definitely yes (low risk of bias)	Definitely yes (low risk of bias)	Definitely yes (low risk of bias)	Definitely yes (low risk of bias)
Wu et al. [68]	2018	Probably no	Probably no	Probably no	Definitely no (high risk of bias)	Probably no	Probably no	Probably no	Definitely yes (low risk of bias)	Definitely yes (low risk of bias)	Probably yes
Yaylali et al. [69]	2004	Probably no	Probably no	Definitely yes (low risk of bias)	Definitely yes (low risk of bias)	Definitely yes (low risk of bias)	Definitely yes (low risk of bias)	Definitely yes (low risk of bias)	Definitely yes (low risk of bias)	Definitely yes (low risk of bias)	Probably yes
Zebardast et al. [70]	2020	Probably no	Probably no	Definitely yes (low risk of bias)	Definitely no (high risk of bias)	Definitely no (high risk of bias)	Definitely no (high risk of bias)	Definitely no (high risk of bias)	Probably yes	Definitely yes (low risk of bias)	Probably yes
Ziakas et al. [71]	2009	Probably no	Probably no	Probably no	Definitely no (high risk of bias)	Definitely yes (low risk of bias)	Definitely yes (low risk of bias)	Definitely yes (low risk of bias)	Definitely yes (low risk of bias)	Definitely no (high risk of bias)	Definitely yes (low risk of bias)

**Table A3:** Surgical parameters for included studies.

Author	Year	Baseline IOP (mmHg), Mean (SD; Range), n Eyes	Post-op IOP (mmHg), Mean (SD); Min:25th: 50th:75th:Max Percentile	OVD Used (n Eyes)	Surgical Technique Performed	Method of IOP Measurement	Clear Corneal Thickness Measured (Yes/No)
Akbari et al. [15]	2021	16.2 (2.9) n = 88	1-day: 22.5 (5.6) n = 88 3-days: 17.5 (2.2) n = 88	-	2.8 mm temporal incision and 5–6 mm capsulorrhexis	Goldmann Applanation Tonometry	No
Akmaz et al. [16]	2023	13.9 (1.5) n = 32 14.2 (2.1) n = 33	1-day: 18.1 (1.4) n = 32 15.4 (3.2) n = 33 7-days: 14.5 (1.4) n = 32 14.1 (2.8) n = 33	1.4% Sodium Hyaluronate (32); None (33)	2.8 mm incision and 5.0–5.5 mm capsulorhexis	Goldmann Applanation Tonometry	Yes
Arshinoff et al. [17]	2002	15.0 (2.4) n = 49 15.2 (2.3) n = 99 15.6 (2.4) n = 50	6 h: 17.6 (4.0) n = 49 18.1 (5.4) n = 99 18.2 (5.2) n = 50 1-day: 17.9 (4.7) n = 49 18.6 (5.3) n = 99 20.2 (6.2) n = 50 7-days: 14.4 (3.2) n = 49 14.3 (3.3) n = 99 14.1 (3.5) n = 50	1.0% Sodium Hyaluronate (49); 1.4% Sodium Hyaluronate (99); 2.3% Sodium Hyaluronate (50)	2.8 mm incision	Goldmann Applanation Tonometry	Yes
Asano et al. [18]	2008	13.9 (3.0) n = 142	1-day: 14.3 (4.1) n = 142 11.6 (3.2) n = 142	-	Small-incision	-	No
Auffarth et al. [19]	2017	15.3 (2.4) n = 111 15.2 (2.7) n = 109	6 h: 20.2 (6.6) n = 111 19.0 (6.7) n = 109 1-day: 16.0 (4.6) n = 111 15.2 (4.6) n = 109 7-days: 14.3 (2.8) n = 111 14.6 (4.4) n = 109	4.0% Chondroitin sulphate- 1.65% sodium hyaluronate (111); 2.2% Sodium hyaluronate—1.0% Sodium hyaluronate (109)	Self-sealing clear corneal incisions	Goldmann Applanation Tonometry	Yes
Balsak et al. [20]	2018	13.9 (3.2) n = 277	6 h: 24.0 (7.4) n = 277 1-day: 15.7 (5.0) n = 277 3-days:13.7 (4.0) n = 277	1.8% Sodium hyaluronate (277)	2.75 mm clear corneal incision	Non-Contact Tonomtery	No
Borazan et al. [21]	2007	14.7 (2.3) n = 185	6 h: 19.1 (5.4) n = 185 1-day: 13.9 (3.9) n = 185 7-days: 14.3 (1.6) n = 185	4.0% Chondroitin sulphate-3.0% sodium hyaluronate (185)	3.2 mm clear corneal temporal incision, 5.0 mm capsulorhexis	Goldmann Applanation Tonometry	No
Camesaca et al. [22]	2007	15.6 (2.7) n = 286	1-day: 15.3 (4.5) n = 286 14.6 (5.2) n = 286	Sodium hyaluronate 3.0%, 1.0% sodium hyaluronate, 4.0% chondroitin sulfate-3.0% sodium hyaluronate, 1.4% sodium hyaluronate, 2.3% sodium hyaluronate, or 1.8% sodium hyaluronate (286)	Temporal incision	Goldmann Applanation Tonometry	No
Cekic et al. [23]	1999	15.8 (3.0) n = 51	8 h: 15.7 (6.1) n = 51 1-day: 15.0 (4.8) n = 51 7-days: 14.4 (3.9) n = 51	Sodium hyaluronate (51)	A clear corneal, curvilinear two-plane two-level incision	Goldmann Applanation Tonometry	No
Cekic et al. [24]	1999	15.1 (3.0) n = 58	1-day: 11.2 (2.4) n = 58 7-days: 11.5 (2.4) n = 58	Chondroitin sulfate-sodium hyaluronate (58)	A clear corneal, curvilinear two-plane two-level incision, with either a 4.0 mm or 6.0 mm capsulorhexis	Goldmann Applanation Tonometry	No
Chang et al. [25]	2017	15.5 (14–19) n = 43	1-day: 18.8 (14–24) n = 43 21-days: 15.0 (13–17) n = 43	1.5% Sodium hyaluronate (43)	2.2 mm clear corneal incision with either Standard or Low fluid settings	Goldmann Applanation Tonometry	Yes
Dieleman et al. [26]	2011	16.1 (3.2) n = 400	1-day: 15.5 (4.6) n = 400 30-days: 14.0 (2.9) n = 400	2.0% Hydroxypropyl methylcellulose (400)	2.8 mm corneal or corneoscleral incision	Goldmann Applanation Tonometry	No
Dieleman et al. [27]	2012	16.0 (3.2) n = 400	1-day: 15.5 (4.6) n = 400 30-days: 14.0 (2.9) n = 400	-	-	Goldmann Applanation Tonometry	No
el-Harazi et al. [28]	1998	17.2 (1.7) n = 58	1-day: 12.4 (2.6) n = 58 7-days: 15.3 (2.3) n = 58	-	5 mm incision	Goldmann Applanation Tonometry	No
Fernandez-Barrientos et al. [29]	2010	23.6 (1.5) n = 16	1-day: 26.4 (8.1) n = 16 14-days: 18.2 (4.2) n = 16	1.0% Sodium hyaluronate (16)	2.8-mm temporal corneal incision	Goldmann Applanation Tonometry	No
Gungor et al. [30]	2014	14.9 (10–17) n = 60	1-day: 17.7 (12–24) n = 60 7-days: 14.3 (11–18) n = 60	4.0% Chondroitin sulphate-3.0% sodium hyaluronate and Sodium hyaluronate 1.0% (60)	2.8 mm clear corneal incision and 5.0 mm capsulorhexis	Goldmann Applanation Tonometry	Yes
Haripriya et al. [31]	2019	14.3 (3.2) n = 1406	1-day: 13.6 (4.8) n = 1406 30-days: 13.6 (3.4) n = 1406	-	2.8 mm clear corneal temporal incision and 5 to 5.5 mm capsulorhexis	Goldmann Applanation Tonometry	No
Holzer et al. [32]	2001	16.0 (3.3) n = 19 16.6 (3.1) n = 30 14.5 (2.7) n = 12 15.4 (3.0) n = 20	6 h: 21.6 (4.5) n = 19 21.8 (7.1) n = 30 24.9 (7.1) n = 12 23.6 (7.5) n = 20 1-day: 17.1 (7.8) n = 19 19.3 (3.8) n = 30 15.7 (4.1) n = 12 17.1 (7.8) n = 20 7-days: 13.3 (2.6) n = 19 13.4 (3.4) n = 30 13.5 (2.6) n = 12 15.5 (3.5) n = 20	1.4% Sodium hyaluronate (19); 2.0% Hydroxypropyl methylcellulose (30); 2.3% Sodium hyaluronate (12); 4.0% Chondroitin sulphate-3.0% sodium hyaluronate (20)	2.5 mm corneoscleral superior incision	Goldmann Applanation Tonometry	No
Holzer et al. [33]	2002	15.6 (3.3) n = 60	1-day: 14.5 (5.4) n = 60 3-days: 13.1 (3.7) n = 60	-	Clear corneal temporal incision	-	No
Honda et al. [34]	2004	13.8 (2.7) n = 44	1-day: 16.5 (5.5) n = 44 13.2 (3.7) n = 44	-	Corneoscleral incision and continuous curvilinear capsulorhexis	-	No
Karalezli et al. [35]	2008	14.8 (2.4) n = 60	1-day: 15.6 (2.4) n = 60 7-days: 14.3 (1.4) n = 60	4.0% Chondroitin sulphate-3.0% sodium hyaluronate and 1.2% sodium hyaluronate (60)	Traditional Phacoemulsification	Goldmann Applanation Tonometry	No
Karalezli et al. [36]	2010	14.0 (2.0) n = 120	6 h: 17.9 (3.0) n = 120 1-day: 16.3 (2.7) n = 120 7-days: 14.0 (1.9) n = 120	4.0% Chondroitin sulphate-3.0% sodium hyaluronate (120)	3.2 mm clear corneal temporal incision and 5.0 mm capsulorhexis	Goldmann Applanation Tonometry	No
Kim et al. [37]	1996	14.5 (2.6) n = 24	1-day: 14.4 (3.4) n = 24 3-days: 14.0 (2.5) n = 24	1.4% Sodium hyaluronate (24)	Traditional Phacoemulsification	Pneumo-tonometry	No
Kohnen et al. [38]	1996	14.8 (2.3) n = 30 14.2 (2.2) n = 30	6 h: 16.8 (3.3) n = 30 16.2 (5.4) n = 30 1-day: 17.7 (3.7) n = 30 17.5 (6.8) n = 30 1.5-days: 15.6 (3.0) n = 30 14.3 (2.3) n = 30 2-days: 15.5 (3.4) n = 30 14.3 (2.5) n = 30 30-days: 14.6 (2.6) n = 30 14.1 (2.8) n = 30	1.0% sodium hyaluronate (30); 1.4% sodium hyaluronate (30)	Traditional Phacoemulsification	Goldmann Applanation Tonometry	No
Kozera et al. [39]	2020	21.9 (2.3) n = 36	1-day: 17.8 (4.3) n = 36 7-days: 16.8 (3.6) n = 36	-	Traditional Phacoemulsification	Goldmann Applanation Tonometry	Yes
Levkovitch-Verbin et al. [40]	2008	15.7 (1.4) n = 122	4 h: 18.0 (4.3) n = 122 8 h: 17.0 (2.7) n = 122 1-day: 15.0 (2.1) n = 122 7-days: 13.9 (1.3) n = 122	1.2% Sodium hyaluronate (122)	Traditional Phacoemulsification	Goldmann Applanation Tonometry	No
Li et al. [41]	2008	16.7 (1.4) n = 70 16.5 (1.4) n = 70	1-day: 18.8 (1.0) n = 70 19.4 (1.2) n = 70 7-days: 12.4 (0.7) n = 70 13.0 (1.0) n = 70	0.1% chitosan (70); 1.4% Sodium hyaluronate (70)	-	Goldmann Applanation Tonometry	Yes
Mahajan et al. [42]	2021	15.9 (2.6) n = 150	1-day: 17.3 (5.8) n = 150 7-days: 15.8 (4.2) n = 150	-	-	Non-Contact Tonometry	No
Malecaze et al. [43]	2000	14.9 (1.0) n = 50	1-day: 14.9 (1.2) n = 50 7-days: 14.7 (1.1) n = 50	1.4% Sodium hyaluronate (50)	3.2 mm clear corneal incision	Goldmann Applanation Tonometry	No
Mastropasqua et al. [44]	1998	16.0 (2.0) n = 100	6 h: 19.9 (4.5) n = 100 1-day: 19.2 (4.7) n = 100 7-days: 16.4 (2.4) n = 100	1.4% Sodium hyaluronate (100)	3.2 mm clear corneal superior incision and 5 mm capsulorhexis	Goldmann Applanation Tonometry	No
Paganelli et al. [45]	2004	14.2 (10–19) n = 100	1-day: 17.3 (12–24) n = 100 3-days: 15.4 (10–22) n = 100	-	2.75-mm clear corneal incision	Goldmann Applanation Tonometry	No
Paganelli et al. [46]	2009	14.0 (3.1) n = 135	1-day: 14.0 (3.9) n = 135 3-days: 13.2 (3.0) n = 132	-	2.75 mm clear corneal incision	Goldmann Applanation Tonometry	No
Probst et al. [47]	1993	14.7 (2.0) n = 25 17.1 (0.6) n = 25	1-day: 20.9 (2.0) n = 25 18.6 (2.0) n = 25 7-days: 15.4 (0.9) n = 25 13.4 (0.8) n = 25	1.6% Sodium hyaluronate (25); 4.0% Chondroitin sulphate-3.0% sodium hyaluronate (25)	Superior limbal incision	-	Yes
Rabsilber et al. [48]	2007	14.8 (2.8) n = 34	1-day: 11.5 (2.7) n = 34 30-days: 11.9 (2.7) n = 34	1.4% Sodium hyaluronate (34)	Sealed capsule irrigation or Traditional	-	Yes
Rainer et al. [49]	2000	14.8 (2.4) n = 50	6 h: 16.8 (4.1) n = 50 1-day: 14.4 (4.2) n = 50 7-days: 13.7 (2.2) n = 50	1.0% Sodium hyaluronate (50)	6.0 mm superior sutureless frown incision	Goldmann Applanation Tonometry	No
Rainer et al. [50]	2000	14.9 (2.5) n = 35 15.2 (2.9) n = 35	6 h: 20.1 (6.2) n = 35 25.2 (9.0) n = 35 1-day: 15.3 (3.9) n = 35 15.0 (3.4) n = 35 7-days: 13.5 (2.3) n = 35 13.5 (2.5) n = 35	2.3% Sodium hyaluronate (35); 4.0% Chondroitin sulphate-3.0% sodium hyaluronate (35)	3.5 mm temporal incision	Goldmann Applanation Tonometry	No
Rainer et al. [51]	2001	14.3 (2.6) n = 80	6 h: 20.6 (7.7) n = 80 1-day: 14.4 (3.8) n = 80 7-days: 13.7 (2.8) n = 80	1.0% Sodium hyaluronate (80)	3.2 mm temporal incision	Goldmann Applanation Tonometry	No
Rainer et al. [52]	2001	13.9 (3.0) n = 60	6 h: 15.3 (4.4) n = 60 1-day: 12.8 (4.0) n = 60 7-days: 13.1 (3.0) n = 60	1.0% Sodium hyaluronate (60)	3.5 mm temporal incision	Goldmann Applanation Tonometry	No
Rainer et al. [53]	2001	14.0 (2.8) n = 40 14.1 (2.8) n = 40	6 h: 19.0 (5.8) n = 40 18.8 (6.0) n = 40 1-day: 15.9 (3.9) n = 40 15.7 (4.7) n = 40 7-days: 13.8 (3.0) n = 40 13.6 (2.6) n = 40	2.0% Hydroxypropyl methylcellulose (40); 4.0% Chondroitin sulphate-3.0% sodium hyaluronate (40)	3.5 mm temporal incision	Goldmann Applanation Tonometry	No
Rainer et al. [54]	2003	15.4 (2.5) n = 76	6 h: 21.8 (6.5) n = 76 1-day: 14.8 (4.2) n = 76 7-days: 14.5 (3.2) n = 76	4.0% Chondroitin sulphate-3.0% sodium hyaluronate (76)	3.5 mm temporal incision	Goldmann Applanation Tonometry	No
Saari et al. [55]	2006	17.9 (3.0) n = 20	1-day: 15.7 (5.5) n = 20 3-days: 14.6 (4.5) n = 20	1.0% Sodium hyaluronate (20)	3.5 mm sclerotunnel superior incision and 5.0 mm capsulorhexis	Goldmann Applanation Tonometry	No
Sandoval et al. [56]	2006	15.1 (3.5) n = 40	1-day: 16.6 (5.2) n = 40 3-days: 12.7 (1.9) n = 40	-	Traditional Phacoemulsification	-	No
Schwenn et al. [57]	2000	13.5 (2.0) n = 20 14.6 (3.0) n = 28	4 h: 18.1 (6.7) n = 20 27.5 (10.1) n = 28 6 h: 16.4 (5.1) n = 20 23.1 (8.5) n = 28 1-day: 12.3 (3.0) n = 20 14.5 (5.5) n = 28 2-days: 9.6 (2.2) n = 20 12.3 (3.5) n = 28 3-days: 9.8 (2.2) n = 20 12.2 (3.8) n = 28	2.3% Sodium hyaluronate (20); 4.0% Chondroitin sulphate-3.0% sodium hyaluronate (28)	3.2 mm inverse curved superior incision	-	Yes
Schwenn et al. [58]	2001	14.4 (2.6) n = 100	6 h: 20.9 (7.6) n = 100 1-day: 15.7 (4.9) n = 100 2-days: 13.5 (3.1) n = 100 3-days: 13.4 (3.0) n = 100	2.0% Hydroxypropyl methylcellulose (100)	Temporal incision being either clear corneal or sclerocorneal incision	Goldmann Applanation Tonometry	No
Simaroj et al. [59]	2011	13.9 (2.2) n = 60	1-day: 13.1 (2.4) n = 60 7-days: 12.6 (2.0) n = 60	-	3-mm clear corneal incision	Goldmann Applanation Tonometry	No
Sipos et al. [60]	2011	16.4 (2.4) n = 60	1-day: 17.0 (1.6) n = 60 7-days: 15.2 (1.6) n = 60	-	-	-	No
Solomon et al. [61]	1998	15.3 (2.6) n = 41	6 h: 18.1 (7.1) n = 41 1-day: 16.3 (3.9) n = 41 7-days: 13.9 (2.6) n = 41	1.4% Sodium hyaluronate (41)	Clear corneal incision	Goldmann Applanation Tonometry	Yes
Stifter et al. [62]	2007	13.4 (2.5) n = 100	1 h: 19.0 (3.3) n = 100 2 h: 18.4 (2.8) n = 100 4 h:16.8 (2.4) n = 100 6 h: 15.8 (2.7) n = 100 8 h: 14.9 (2.8) n = 100 1-day: 14.2 (2.6) n = 100 7-days: 14.4 (2.1) n = 100	1.0% Sodium hyaluronate (100)	Combined Primary posterior continuous curvilinear capsulorhexis and posterior optic buttonholing or Conventional in-the-bag IOL implantation	Goldmann Applanation Tonometry	No
Stifter et al. [63]	2010	13.8 (1.9) n = 60	1 h: 20.8 (6.7) n = 60 2 h: 19.4 (5.9) n = 60 4 h: 18.5 (5.2) n = 60 6 h: 17.6 (4.5) n = 60 8 h: 16.5 (3.1) n = 60 1-day: 15.5 (2.4) n = 60 7-days: 14.1 (2.2) n = 60	1.0% Sodium hyaluronate and 2.0% Hydroxypropyl methylcellulose (60)	Combined Primary posterior capsulorhexis and posterior optic buttonholing or Conventional in-the-bag IOL implantation	Goldmann Applanation Tonometry	No
Studeny et al. [64]	2014	16.8 (3.9) n = 96 16.4 (3.9) n = 96	2 h: 10.2 (6.8) n = 96 9.9 (7.0) n = 96 1-day: 14.5 (5.6) n = 96 15.5 (5.8) n = 96 30-days: 13.2 (3.5) n = 96 13.1 (3.4) n = 96	2.0% Hydroxypropyl methylcellulose (96); 1.0% Sodium hyaluronate and 2.0% Hydroxypropyl methylcellulose (96)	Either hydroimplantation of IOL or OVD use in IOL implantation	Non-Contact Tonometry	No
Waseem et al. [65]	2007	14.3 (2) n = 46 14.1 (1.9) n = 45	1-day: 18.8 (2.9) n = 46 16.9 (2.6) n = 45 7-days: 14.7 (1.6) n = 46 14.4 (1.7) n = 45	1.0% Sodium hyaluronate (46); 2.0% Hydroxypropyl methylcellulose (45)	3.2 mm temporal limbal incision	Goldmann Applanation Tonometry	No
Wedrich et al. [66]	1992	14.2 (3.2) n = 90	6 h: 15.1 (8.2) n = 90 18 h: 14.3 (6.5) n = 90 7-days: 13.8 (2.5) n = 90	1.0% Sodium hyaluronate (90)	Traditional Phacoemulsification	Goldmann Applanation Tonometry	No
Wirtitsch et al. [67]	2007	16.6 (2.6) n = 88	6 h: 18.7 (5.4) n = 88 1-day: 15.2 (3.5) n = 88 7-days: 16.0 (2.9) n = 88	1.0% Sodium hyaluronate (88)	Traditional Phacoemulsification	Goldmann Applanation Tonometry	No
Wu et al. [68]	2018	14.5 (2.6) n = 99	1-day: 15.2 (5.0) n = 99 7-days: 14.6 (3.3) n = 99	1.0% Sodium hyaluronate (99)	Standard 3.0 mm small incision phacoemulsification or Coaxial 2.2 mm micro-incision Fusion Phaco complex phacoemulsification	Non-Contact Tonometry	Yes
Yaylali et al. [69]	2004	13.3 (2.5) n = 48	1-day: 14.7 (5.6) n = 48 3-days: 11.3 (1.7) n = 48	4.0% Chondroitin sulphate-3.0% sodium hyaluronate and 1.2% Sodium hyaluronate (48)	Traditional Phacoemulsification	Goldmann Applanation Tonometry	No
Zebardast et al. [70]	2020	25.4 (2.9) n = 187	1-day: 27.6 (10.2) n = 187 7-days: 20.8 (6.3) n = 187	-	Traditional Phacoemulsification	Goldmann Applanation Tonometry	Yes
Ziakas et al. [71]	2009	14.5 (3.0) n = 30	1-day: 12.7 (4.5) n = 30 7-days: 13.2 (2.3) n = 30	Sodium hyaluronate (30)	Traditional Phacoemulsification	Goldmann Applanation Tonometry	No

**Table A4:** Medications administered for included studies.

Author	Year	Pre-Operative Medications (n Eyes)	Intra-Operative Medications (n Eyes)	Post-Operative Medications (n Eyes)
Akbari et al. [15]	2021	None	None	Chloramphenicol 5%, betamethasone 0.1%, and ketorolac tomethamine 0.5%(46) Or Chloramphenicol 5%, and betamethasone 0.1% (42)
Akmaz et al. [16]	2023	None	0.1 cc moxifloxacin (65)	None
Arshinoff et al. [17]	2002	Ofloxacin 0.3%, and diclofenac 0.1% eyedrops (198)	None	Tobramycin and dexamethasone eyedrops (198)
Asano et al. [18]	2008	Diclofenac sodium 0.1% (71) or Betamethasone sodium 0.1% (71)	None	Diclofenac sodium 0.1% (71) Or Betamethasone sodium 0.1% (71)
Auffarth et al. [19]	2017	-	-	-
Balsak et al. [20]	2018	None	Cefurozime axetil 0.1% (277)	Brimonidine-timolol, tobramycin and dexamethasone (231) Or Tobramycin and dexamethasone (46)
Borazan et al. [21]	2007	Diclofenac (185)	None (153) or Carbachol (32)	Acetazolamide 250 mg, ofloxacin 0.3% and prednisolone acetate 1% eyedrops (185) With either Topical brinzolamide 1% (30), Brimonidine 0.2% (32), Oral acetazolamide 250 mg (30), Timolol maleate 0.5% (30), Or nothing else (63)
Camesaca et al. [22]	2007	Fluoroquinolone (286)	None	Chloramphenicol 0.25% and betamethasone 0.13% gel (143) Or Tobramycin 0.3% and dexamethasone 0.1% (143)
Cekic et al. [23]	1999	None	None	0.5 mL balanced salt solution (24) Or 0.5 mL carbachol 0.01% (27)
Cekic et al. [24]	1999	None	Carbachol (58)	20 mg gentamicin and 2 mg betamethasone (58)
Chang et al. [25]	2017	None	0.2 mg moxifloxacin (43)	Dexamethasone (43)
Dieleman et al. [26]	2011	None	None	Betamethasone acetate 5.7 mg/mL (200) Or Dexamethasone 0.1% (200)
Dieleman et al. [27]	2012	None	None	Physostigmine, and dexamethasone (199) Or Dexamethasone (201)
el-Harazi et al. [28]	1998	None	None	Diclofenac sodium 0.1% (19) Or Ketorolac tromethamine 0.5% (19) Or Prednisolone acetate 1% (20)
Fernandez-Barrientos et al. [29]	2010	1 mg/mL tetracaine chloride and 4mg/mL oxybuprocainechloride (16)	None	None
Gungor et al. [30]	2014	None	None	Triamcinolone acetonide 2 mg/0.05 mL (30) Or Dexamethasone (0.4 mg/0.1 mL (30)
Haripriya et al. [31]	2019	Topical ofloxacin 0.3% (1406)	None	Topical ofloxacin 0.3% and prednisolone acetate 1% (1406)
Holzer et al. [32]	2001	-	-	-
Holzer et al. [33]	2002	Ofloxacin 0.3% (60)	Epinephrine and carbachol (60)	Loteprednol etabonate 0.5% and ofloxacin 0.3% (30) Or Ketorolac tromethamine 0.5% and ofloxacin 0.3% (30)
Honda et al. [34]	2004	-	-	Topical mixture of unspecified antibiotics and steroids (44)
Karalezli et al. [35]	2008	None	None (30) Or 1 mg intracameral triamcinolone acetonide (30)	Topical prednisolone acetate 1% and topical ofloxacin 0.3% (30) Or Topical ofloxacin 0.3% (30)
Karalezli et al. [36]	2010	None	None	None (60) Or 1mg triamcinolone acetonide (60)
Kim et al. [37]	1996	Tobramycin (24)	None	Topical 0.1% fluorometholone (24)
Kohnen et al. [38]	1996	Unspecified antibiotics eye drops and oral 250 mg acetazolamide (60)	None	Dexamethasone, polymyxin, neomycin, and unspecified steroid/antibiotic drops (60)
Kozera et al. [39]	2020	None	None	Loteprednol and Moxifloksacinum (36)
Levkovitch-Verbin et al. [40]	2008	Unspecified anti-glaucoma medications (33) Or None (89)	None	Dexamethasone 0.1% eye drops and chloramphenicol ointment (122) With either Timolol maleate (60), unspecified anti-glaucoma medications (33), and/or nothing else (44)
Li et al. [41]	2008	-	-	-
Mahajan et al. [42]	2021	-	-	Topical difluprednate 0.05% (50) Or Topical dexamethasone 0.1% (50) Or Topical prednisolone 1% (50)
Malecaze et al. [43]	2000	None	None	Gentamicin eye drops (50)
Mastropasqua et al. [44]	1998	-	None	-
Paganelli et al. [45]	2004	None	None (50) Or 40-mg triamcinolone acetonide (50)	Prednisolone acetate 1% (50) Or None
Paganelli et al. [46]	2009	None	None (67) Or 25 mg triamcinolone and 2 mg ciprofloxacin (68)	Prednisolone acetate 1% and ciprofloxacin hydrochloride 0.3% (67) Or None (68)
Probst et al. [47]	1993	None	None	None (42) Or Topical Beta blocker (8)
Rabsilber et al. [48]	2007	None	None	None
Rainer et al. [49]	2000	Diclofenac (50)	None	Betamethasone-neomycin ointment, diclofenac and prednisolone acetate (50) With either Apraclonidine 0.5% (25) Or dorzolamide 2% (25)
Rainer et al. [50]	2000	Diclofenac (70)	None	Gentamicin-prednisolone ointment, and diclofenac and prednisolone acetate 0.5% eyedrops (70)
Rainer et al. [51]	2001	Diclofenac (80)	None	Prednisolone acetate 0.5% ointment and diclofenac and prednisolone acetate 0.5% eyedrops (80) With either brimonidine 0.2% eye drops (40) Or prednisolone acetate 0.5% ointment (40)
Rainer et al. [52]	2001	Diclofenac eye drops (80)	None	Prednisolone ointment and diclofenac and prednisolone acetate 0.5% eyedrops (80)
Rainer et al. [53]	2001	Diclofenac eyedrops (60)	None	Acetate 0.5% ointment, and diclofenac and prednisolone acetate 0.5% eyedrops (60) With either Prednisolone fixed dorzolamide–timolol combination eye drops (30), Or latanoprost eye drops (30)
Rainer et al. [54]	2003	None	None	None (38) Or Fixed dorzolamide–timolol combination (38)
Saari et al. [55]	2006	250 mg Oral acetazolamide (20)	None	0.7% or 0.1% dexamethasone with chloramphenicol eye drops, and dexamethasone alcohol and neomycin sulphate ointment (20)
Sandoval et al. [56]	2006	Ketorolac 0.4% or 0.5%, eye drops (40)	None	Ketorolac 0.4% or 0.5% and ofloxacin 0.3% (40)
Schwenn et al. [57]	2000	Topical diclofenac (48)	None	Prednisolone acetate eye drops and ointment, and gentamicin eye drops (48)
Schwenn et al. [58]	2001	None	None	Prednisolone acetate eyedrops, gentamicin sulfate, and 5 mg/g prednisolone pivalate ointment (100)
Simaroj et al. [59]	2011	None	None (30) Or Intracameral triamcinolone acetonide and gentamicin (30)	Topical 0.1% dexamethasone-0.3% tobramycin combination (30) Or Topical tobramycin (30)
Sipos et al. [60]	2011	Diclofenac eye drops (60)	None	Diclofenac sodium and gentamicin eyedrops (60) With either Low-viscosity tear replacement eye drops (11), high-viscosity tear replacement eye drops (9), 0.3 mg dexamethasone and 3 mg gentamicin sulfate ointment (10), or nothing else (30)
Solomon et al. [61]	1998	None	None (20) Or Intracameral carbachol 0.01% (21)	Unspecified topical antibiotic-corticosteroid combination (41)
Stifter et al. [62]	2007	None	None	Dexamethasone-gentamicin ointment, diclofenac sodium and prednisolone acetate 0.5% eyedrops (100) With either Dorzolamide hydrochloride 2.0%–timolol maleate 0.5% eye drops (50) Or Nothing else (50)
Stifter et al. [63]	2010	Diclofenac eye drops (60)	None	None
Studeny et al. [64]	2014	None	None	None
Waseem et al. [65]	2007	None	None	Norfloxacin 0.3%, dexamethasone eye 0.1% drops, and Fusidic acid eye ointment (91)
Wedrich et al. [66]	1992	None	None (30) Or Intracameral carbachol (30) Or Intracameral acetylcholine 1%	Topical steroids and indomethacin (90)
Wirtitsch et al. [67]	2007	None	Fixed dorzolamide–timolol combination eye drop (88)	Prednisolone acetate 1% and diclofenac eye drops (88)
Wu et al. [68]	2018	None	None	Levofloxacin and prednisolone eye drops (99)
Yaylali et al. [69]	2004	None	None	Ofloxacin 0.3% (48) With either prednisolone acetate 1% eyedrops (21) Or topical rimexolone 1% (27)
Zebardast et al. [70]	2020	None	None	Unspecified topical antibiotics and corticosteroids (187)
Ziakas et al. [71]	2009	None	None (15) Or Trypan Blue (15)	1 tablet of acetazolamide 250 mg, and dexamethasone 1mg/mL and chloramphenicol 5 mg/mL eye drops (30)
